# Predictors of Progression and Mortality in Patients with Chronic Hypersensitivity Pneumonitis: Retrospective Analysis of Registry of Fibrosing Interstitial Lung Diseases

**DOI:** 10.3390/life13020467

**Published:** 2023-02-08

**Authors:** Natalia V. Trushenko, Olga A. Suvorova, Ekaterina S. Pershina, Galina V. Nekludova, Svetlana Yu. Chikina, Iuliia A. Levina, Andrey L. Chernyaev, Maria V. Samsonova, Igor E. Tyurin, Malika Kh. Mustafina, Andrey I. Yaroshetskiy, Nikita B. Nadtochiy, Zamira M. Merzhoeva, Anna A. Proshkina, Sergey N. Avdeev

**Affiliations:** 1 Pulmonology Department, Sechenov First Moscow State Medical University (Sechenov University), Healthcare Ministry of Russia, Trubetskaya St. 8, Build. 2, 119991 Moscow, Russia; 2 Pulmonology Scientific Research Institute, Federal Medical and Biological Agency of Russian Federation, Orekhovyy Boulevard 28, 115682 Moscow, Russia; 3 Pirogov City Clinical Hospital No. 1, Moscow Healthcare Department, Leninsky Avenue 8, 117049 Moscow, Russia; 4 Morphology Department, Pirogov Federal Russian National Research Medical University, Healthcare Ministry of Russia, Ostrovityanova St. 1, 117997 Moscow, Russia; 5 Federal Research Institute of Human Morphology, Tsyurupy St. 3, 117418 Moscow, Russia; 6 Moscow Clinical Scientific Center, Enthusiasts Highway 84/1, 111123 Moscow, Russia; 7 Russian Federal Academy of Continued Medical Education, Healthcare Ministry of Russia, Barrikadnaya St. 2/1, Build. 1, 123995 Moscow, Russia; 8 Chelyabinsk Regional Clinical Hospital, Vorovskogo St. 70, 454076 Chelyabinsk, Russia; 9 Radiology Department, South-Ural State Medical University, Healthcare Ministry of Russia, Vorovskogo St. 64, 454092 Chelyabinsk, Russia

**Keywords:** hypersensitivity pneumonitis, progressive pulmonary fibrosis, pulmonary fibrosis, interstitial lung diseases, prognosis, mortality, registry

## Abstract

Hypersensitivity pneumonitis (HP) is an interstitial lung disease (ILD) resulting from an immune-mediated response in susceptible and sensitized individuals to a large variety of inhaled antigens. Chronic HP with a fibrotic phenotype is characterized by disease progression and a dismal prognosis. The aim of this study was to identify predictors of progression and mortality in patients with chronic HP in real clinical practice. Materials and methods: This retrospective, multicenter, observational study used data from a registry of 1355 patients with fibrosing ILDs. The study included 292 patients diagnosed with chronic HP based on the conclusion of a multidisciplinary discussion (MDD). Results: The patients were divided into groups with progressive (92 (30.3%) patients) and nonprogressive pulmonary fibrosis (200 (69.7%) patients). The most significant predictors of adverse outcomes were a DLco < 50% predicted, an SpO2 at the end of a six-minute walk test (6-MWT) < 85%, and a GAP score ≥ 4 points. Conclusion: Pulmonary fibrosis and a progressive fibrotic phenotype are common in patients with chronic HP. Early detection of the predictors of an adverse prognosis of chronic HP is necessary for the timely initiation of antifibrotic therapy.

## 1. Introduction

Hypersensitivity pneumonitis (HP) is an immune-mediated interstitial lung disease (ILD) that manifests in individuals susceptible and sensitized to inhaled antigens. HP accounts for about 4–13% of all ILDs [1]. According to the results of recently published studies, the incidence of HP was 1.16 per 100,000 people in Denmark and 0.9 per 100,000 people in the UK [2,3]. At the same time, the presence of HP significantly increased the risk of death in these patients [3]. In a study by Ojanguren et al., five-year mortality from chronic HP was 31.5% [4]. In addition, Fernández Pérez et al. showed that in recent years (from 1988 to 2016) there has been a trend of increasing mortality from HP, from 0.12 per million to 0.68 per million [5].

To date, more than 200 antigens are known that can cause this disease, while the number of etiological HP antigens continues to increase [6,7]. At the same time, in many patients with HP, it is not possible to identify the etiological antigen, despite a careful investigation of the source of exposure [8]. HP is a heterogeneous disease with varying phenotypes and prognoses. The fibrotic phenotype of chronic HP is characterized by a progressive disease trajectory and a worse prognosis compared with patients with nonfibrotic HP [6,7].

Of particular importance are two recently published guidelines for the diagnosis and management of HP [6,9]. Identification of various chronic HP phenotypes (fibrotic and nonfibrotic) are of great practical importance, as they determine the approaches to the treatment of patients.

Patients with a fibrotic HP phenotype are characterized by worsening clinical symptoms, decreased lung function, decreased quality of life, development of respiratory failure, and decreased survival [10,11]. Although the criteria for progressive pulmonary fibrosis are still the subject of intense discussion, there are currently several proposals for such criteria for use in clinical trials and in real clinical practice [12,13]. 

Considering the increasing mortality of HP patients over the past decades [14], there is an increasing interest in identifying predictors of the progression and mortality in HP patients. The aim of this study was to identify predictors of the progression and mortality in patients with chronic HP in real clinical practice.

## 2. Materials and Methods

### 2.1. Study Design

This was a nationwide, multicenter, observational, and noninterventional longitudinal study. We used data from the national registry of 1355 patients with fibrotic ILDs, conducted from 2016 to 2020 with the participation of 73 clinical centers of the Russian Federation [15]. Anamnesis data, clinical characteristics, functional lung tests, and chest high-resolution computed tomography (HRCT) were entered by the attending physicians in a special unified form [15]. All referred ILD patients were reviewed in a multidisciplinary discussion (MDD). The multidisciplinary panel included pulmonologists, radiologists, and, whenever needed, pathologists. The study was performed in accordance with the declaration of Helsinki and its subsequent revisions. The study was approved by the Institutional Review Board of the Pulmonology Research Institute, Moscow (protocol №3-02-16), and written informed consent was obtained from every patient.

### 2.2. Patients

The present study included patients aged 18 years and older with a diagnosis of chronic HP that was made on the basis of the conclusions of the MDD. Exposure factors were taken into account (contact with mold, birds, silage, hay, cutting fluids, isocyanates, etc.), noted in a special column by the attending physicians. Mandatory inclusion criteria were the presence of HRCT patterns of “typical HP” or “compatible with HP” [6].

According to the course of the disease, all patients were divided into two groups: those with the progressive and those with the nonprogressive type. We used the criteria for the progression of pulmonary fibrosis according to the 2022 ATS/ERS/JRS/ALAT Clinical Practice Guidelines [13]. PPF is defined as at least two of the following three criteria occurring within the past year with no alternative explanation:

- Worsening respiratory symptoms;
- Absolute decline in the forced vital capacity (FVC) > 5% predicted or absolute decline in the diffusive capacity of the lungs (DLco) > 10% predicted within 1 year of follow-up;
- Radiological evidence of disease progression.

Only patients with complete follow-up data were included in this study.

### 2.3. Data Collection

The analysis included the patients’ demographic and clinical characteristics: age, gender, time from symptom onset to diagnosis, body mass index, smoking history, mMRC dyspnea score, respiratory rate, heart rate, prevalence of comorbidities, the Charlson comorbidity index, and the 6 min walk test (6-MWT). Blood analysis parameters (including the number of monocytes and the ratio of neutrophils and lymphocytes), the level of C-reactive protein, and the arterial blood gas analysis were taken from the medical records.

Pulmonary function tests (spirometry, body plethysmography, and DLco) were performed as routine measurements in all patients according to the ATS-ERS guidelines and were reported as absolute values and percentages of predicted values. Based on gender, age, FVC, and DLco as predicted percentages, the GAP score was calculated.

Some echocardiographic parameters, such as right heart dimensions, systolic pulmonary arterial pressure (SPAP), right atrium area, and tricuspid annular plane systolic excursion (TAPSE) were also included in the analysis.

The treatment regimens for patients with chronic HP consisted of oral corticosteroids, both in monotherapy and in combination with immunosuppressive drugs (azathioprine or mycophenolate mofetil), and several patients received antifibrotic therapy (nintedanib or pirfenidone). The choice of therapy regimen depended on the decision of the attending physician.

### 2.4. Statistical Analysis

The normality of data distribution was assessed using the Shapiro–Wilk test. Discrete variables are presented as frequencies, and continuous variables are presented as a median with interquartile range (IQR) or a mean with standard deviation (SD). Comparison of the continuous variables between nonfibrotic and fibrotic phenotype groups was performed using the Mann–Whitney U-test. Categorical variables between groups were compared using Fisher’s exact test or Pearson’s chi-square test. We used logistic regression, including multivariate regression analysis, to explore the risk factors associated with progression and death in HP patients. Odds ratios (OR) and 95% confidence intervals (CI) were calculated. Receiver operator characteristic curves (ROC) were used to calculate the sensitivity of and specificity for predicting progression and death by risk factors and to determine the optimal prognostic cutoff value (Youden method). The ROC-curve analysis results are presented as the area under the curve (AUC), diagnostic significance level (p), and 95% CI. Survival curves were obtained using the Kaplan–Meier method and were compared by the log-rank test. Differences were considered statistically significant at p less than 0.05. Statistical data processing was carried out using IBM SPSS Statistics, version 26 software (SPSS, Chicago, IL, USA).

## 3. Results

The study included 292 patients with chronic HP. The average follow-up period for patients was 11 (6–17) months. The main characteristics of patients are presented in Table 1.

The median age of patients with chronic HP was 65 (58–71) years; there was an approximately equal distribution of patients by sex (men 43.8%, women 52.3%). Smokers accounted for 17.9% of the entire group of patients, and the average smoking history was 20 (13–32) packs/years.

Exposure to a possible causal antigen was documented in only 15.1% of patients.

Histopathological evaluation was performed in 77 patients (26.4%).

Among the clinical manifestations of chronic HP, the most common were dry cough (73.4%) and dyspnea (86.5%) (mMRC score 2 (2–3) points). Finger clubbing was presented in 21.6% of patients.

The predominant comorbidities were arterial hypertension (54.6%), other cardiovascular diseases (35.5%), gastroesophageal reflux disease (17.8%), and diabetes mellitus (14.5%). The presence of pulmonary hypertension was noted in 18.1% of patients.

The median FVC was 67.0 (57.0–78.0)% predicted, and the DLco was 51.2(41.6–59.8)% predicted. Patients with the first (52.6%) and second (42.1%) stages of GAP prevailed. At the end of the 6-MWT, there was significant desaturation in most patients: the mean SpO2 at baseline was 95 (92–96)%, and at the end of the test, the mean SpO2 was 86 (81–90)%. Long-term oxygen therapy was received by 61 patients (20.1%).

The main therapy for the treatment of HP was systemic corticosteroids (41.1%); immunosuppressants (4.6%) were used much less frequently. Antifibrotics were administered to 11.5% of patients (however, that registry data was collected until 2020, when these drugs were not yet approved for the treatment of advanced non-IPF pulmonary fibrosis).

Among the included patients with chronic HP, 279 (91.8%) patients had a fibrotic phenotype, and a progressive course was confirmed in 92 (30.3%) patients. During the follow-up, 44 (47.8%) patients died in the group of patients with progressive pulmonary fibrosis, while there were no deaths among patients with a nonprogressive phenotype. The median survival of deceased patients was 9.5 (2.0–20.8) months. The course of the disease significantly differed between the groups of patients with progressive and nonprogressive chronic HP. Differences between patients with progressive and nonprogressive chronic HP are presented in Table 2.

It was found that patients with a progressive course of chronic HP initially had a longer smoking history (30 (19–40) packs/years vs. 20 (9–30) packs/years, *p* = 0.003), a higher GAP score (4 (3–5) vs. 3 (2–4), *p* = 0.02), a higher Charlson comorbidity index (3 (3–5) points vs. 3 (2–4) points, *p* = 0.04), and a higher prevalence of arterial hypertension (66.3% vs. 52.5%, *p* = 0.03) and diabetes mellitus (23.9% vs. 11.0%, *p* = 0.004). Significant differences in functional methods were found only in DLco (44.5 (35.1–53.2)% predicted vs. 55.0 (45.0–65.0)% predicted, *p* = 0.0001) and in SpO2 values at the end of 6-MWT (84 (79–88)% versus 88 (84–90)%, *p* = 0.001).

Patients with a progressive course of HP were more often prescribed antifibrotics (21.7 vs. 9.0%, *p* = 0.01) and immunosuppressants (19.6 vs. 7%, *p* = 0.03). There were no significant differences in the frequency of prescribing SCSs.

During the follow-up period, 44 (47.8%) patients died in the group of patients with progressive pulmonary fibrosis, while there were no deaths among the patients with a nonprogressive phenotype.

### 3.1. Predictors of Progression

The regression analysis revealed relationships between increased risk of disease progression and male sex (OR 1.68 (95% CI 1.02–2.76), *p* = 0.041), Charlson comorbidity index (OR 1.48 (95% CI 1.03–2.12), *p* = 0.036), and smoking status (1.1 (95% CI 1.0–1.1), *p* = 0.002). The baseline DLco and GAP score are also significant factors for HP progression (0.925 (95% CI 0.89–0.97), *p* = 0.001 and OR 1.57 (95% CI 1.1–2.3), *p* = 0.015, respectively). According to the results of multivariate regression analysis, the main predictive factor for HP progression was the DLco % predicted (OR 0.823, 95% CI 0.71–0.96, *p* = 0.03).

**Figure 1:**
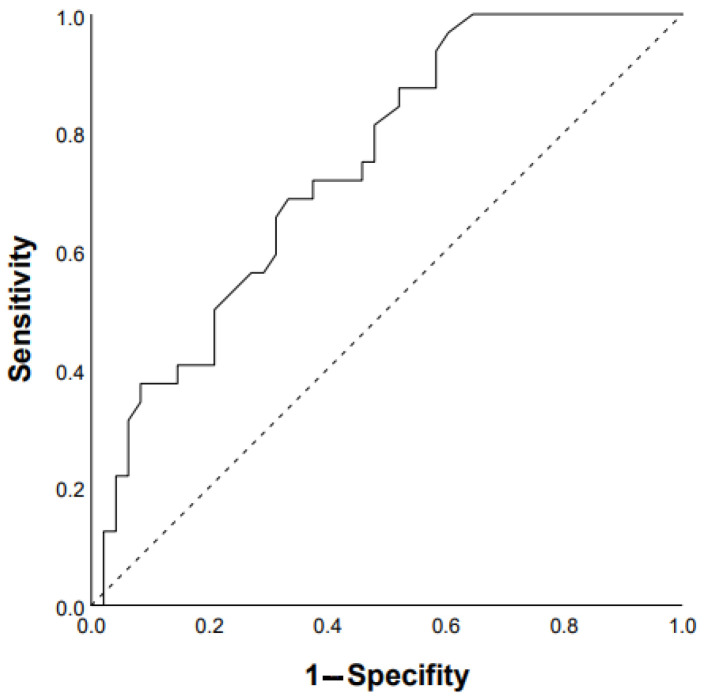
Prediction of chronic HP progression based on DLco < 50% of predicted: AUC 0.742 (95% CI 0.64–0.85), *p* = 0.0001 (ROC curve).

**Figure 2:**
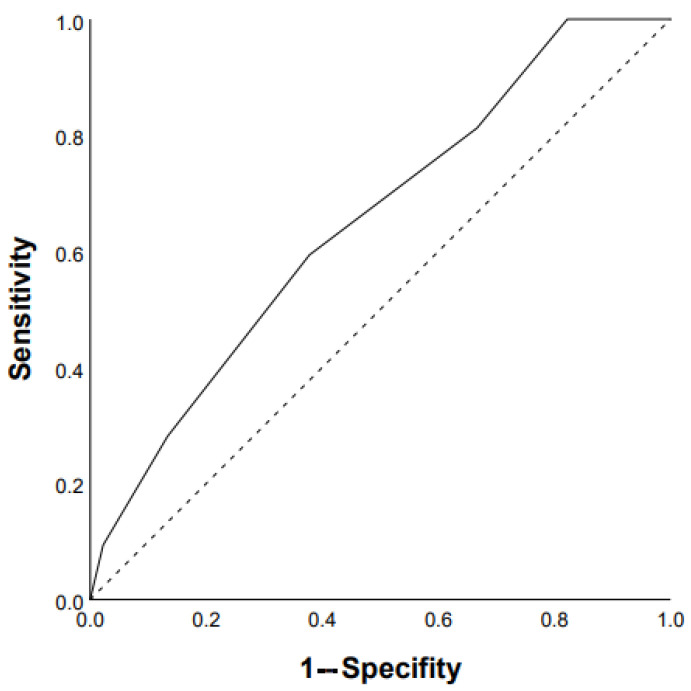
Prediction of chronic HP progression based on GAP score ≥4 points: AUC 0.651 (95% CI 0.53–0.77), *p* = 0.03 (ROC curve).

**Figure 3:**
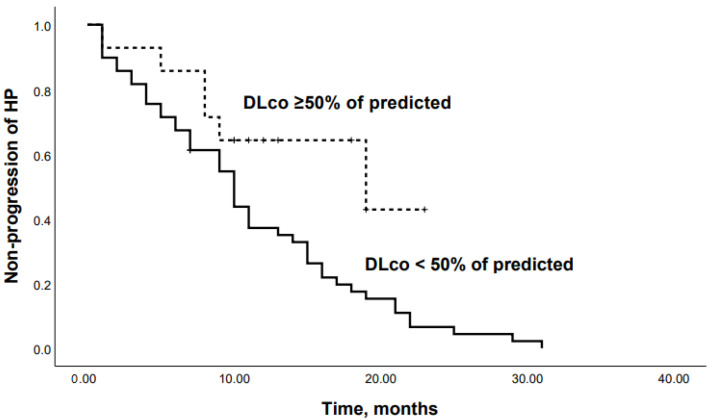
Kaplan–Meier curves: progression in chronic HP patients with DLco <50% and DLco ≥50% of predicted (*p* = 0.04).

ROC analysis showed that the DLco % predicted and GAP scale can serve as a tool to predict the progression of chronic HP: DLco < 50% of predicted (Se 68.8%, Sp 66.7%, area under the curve (AUC) 0.742 (95% CI 0.64–0.85; *p* = 0.0001)) (Figure 1); GAP score ≥ 4 points (Se 59.4%, Sp 62.2%, AUC 0.651 (95% CI 0.53–0.77); *p* = 0.03) (Figure 2); and Charlson comorbidity index ≥ 3 points (Se 81.3%, Sp42.4%, AUC 0.647 (95% CI 0.51–0.72), *p* = 0.04). Kaplan–Meier analysis showed significant differences in the progression of HP in the groups of patients stratified by DLco < 50% predicted (*p* = 0.04) (Figure 3).

### 3.2. Predictors of Mortality

Regression analysis revealed significant relationships between an increased risk of mortality and baseline mMRC dyspnea (OR 2.1, 95% CI 1.4–3.1; *p* = 0.0001), finger clubbing (OR 2.2, 95% CI 1, 1–4.4; *p* = 0.023), initial SpO2 in 6-MWT (OR 0.92, 95% CI 0.87–0.98; *p* = 0.005), SpO2 at the end of 6-MWT (OR 0.86, 95% CI 0.79–0.93, *p* = 0.0001), PaO2 (OR 0.95, 95% CI 0.89–0.99; *p* = 0.04), FVC % predicted (0.96, 95% CI 0.93–0.99; *p* = 0.01), DLco % predicted (OR 0.93, 95% CI 0.88–0.99; *p* = 0.02), right atrial (RA) area (OR 1.35, 95% CI 1.034–1.767, *p*= 0.028), pulmonary hypertension (OR 2.6, 95% CI 1.3–5.4; *p* = 0.007), and diabetes mellitus (OR 3.3, 95% CI 1.6–6.9; *p* = 0.03). 

In a final model, only DLco emerged as an independent predictor of mortality after adjustment in a multivariable analysis (OR 0.823, 95% CI 0.71–0.96, p = 0.03). The influence of the RA area was statistically insignificant (OR 0.864, 95% CI 0.521–1.433, *p* = 0.051), probably due to insufficient data for this parameter.

**Figure 4:**
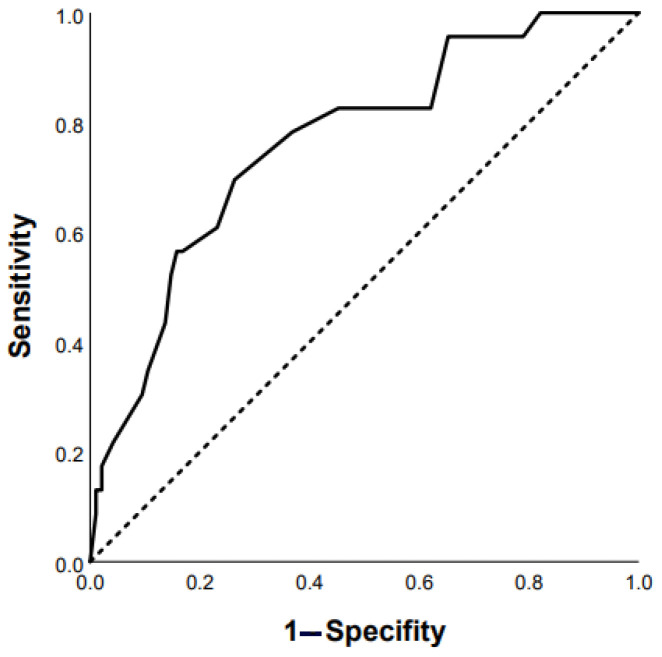
Prediction of mortality in chronic HP patients based on post-SpO2 < 85% in 6-MWT: AUC 0.763 (95% CI 0.66–0.87), *p* = 0.0001 (ROC curve).

**Figure 5:**
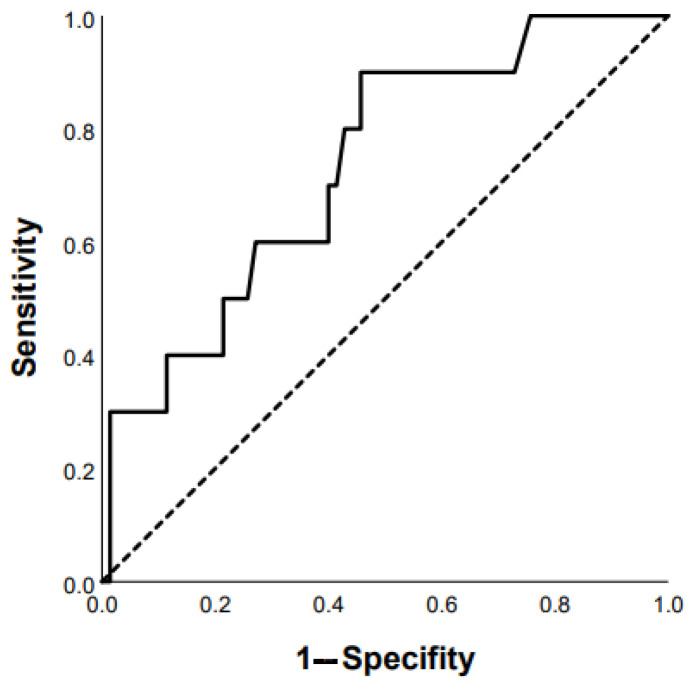
Prediction of mortality in chronic HP patients based on DLco < 50% of predicted: AUC 0.734 (95% CI 0.58–0.89), *p* = 0.02 (ROC curve).

ROC analysis revealed that the following parameters can be used as prognostic factors for the prediction of mortality: RA area ≥ 18 mm^2^ (Se 47.4%, Sp 70.1% AUC 0.684 (95% CI 0.562–0.805), *p* = 0.012); mMRC ≥ 3 points (Se 73.2%, Sp 55.1% AUC 0.685 (95% CI 0.597–0.772), *p* = 0.0001); FVC ≥ 65 % predicted (Se 63.3%, Sp 58.6% AUC 0.659 (95%CI 0.554–0.764), *p* = 0.006); presence of diabetes mellitus (AUC 0.598 (95% CI 0.500–0.696), *p* = 0.04); and finger clubbing (AUC 0.598 (95% CI 0.503–0.693), *p* = 0.05). In addition, there were statistically significant prognostic factors such as SpO2 at the end of 6-MWT < 85% and DLco < 50% predicted (Figure 4 and Figure 5): Se 74%, Sp 68%, AUC 0.763 (95% CI 0.66–0.87; *p* = 0.0001) and Se 80%, Sp 57%, AUC 0.734 (95% CI 0.58–0.89; *p* = 0.02), respectively.

**Figure 6:**
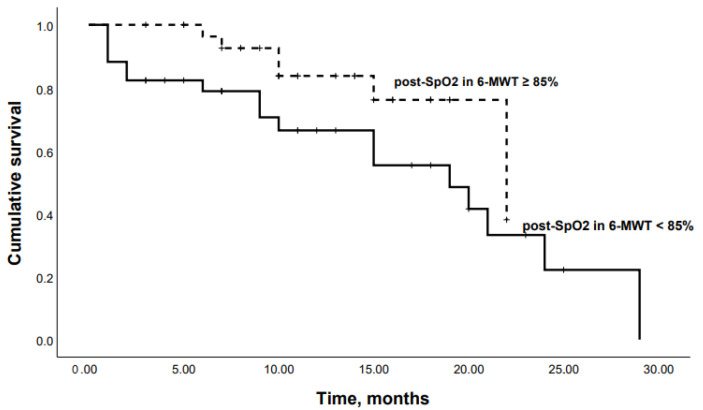
Kaplan–Meier curves: mortality in chronic HP patients with SpO2 at the end of 6-MWT < 85% and SpO2 at the end of 6-MWT ≥ 85% (*p* = 0.04).

**Figure 7:**
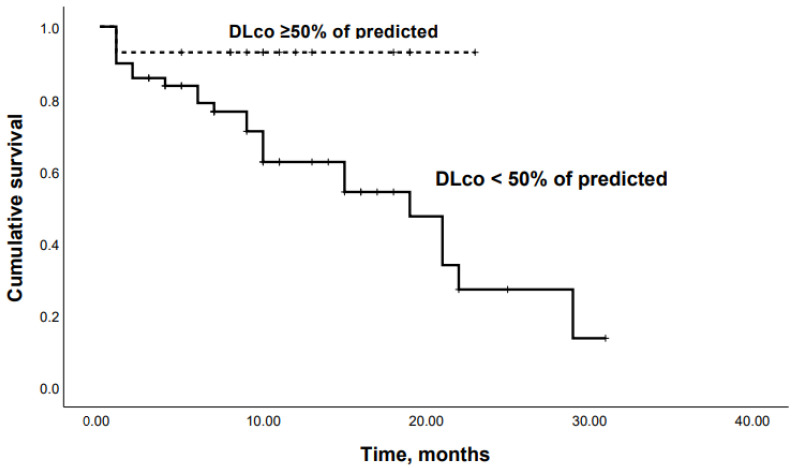
Kaplan–Meier curves: mortality in chronic HP patients with DLco < 50% and DLco ≥50% of predicted (*p* = 0.03).

When patients were dichotomized according to the Youden’s Index for an optimal cutoff point from the ROC curve, Kaplan–Meier analysis revealed that patients with an SpO2 at the end of 6-MWT < 85% or a DLco < 50% had significantly lower survival rates (*p* = 0.04 and *p* = 0.03, respectively) (Figure 6 and Figure 7).

## 4. Discussion

Our retrospective, multicenter, observational study, based on data from a large registry of patients with fibrosing ILDs, included 292 patients with chronic HP, and we showed that 30.3% of patients had a progressive fibrotic phenotype. The most significant predictors of progression and mortality were a baseline DLco <50% predicted, an SpO2 at the end of 6-MWT < 85%, an RA area ≥ 18 mm^2^, and a GAP score ≥4 points.

It should be noted that the mortality rate in patients with fibrotic HP can be comparable to that in patients with IPF [16]. Hanak et al. showed that the presence of signs of fibrosis on HRCT in patients with chronic HP was associated with a significant increase in mortality (OR 4.6, 95% CI 2.0–20.1) [17]. The course of progressive pulmonary fibrosis is comparable to that of IPF, which was confirmed by a comparison of post hoc studies of INPULSIS and INBUILD data [18]. The presence of a typical usual interstitial pneumonia (UIP) pattern on HRCT had a significant impact on the prognosis in patients with progressive pulmonary fibrosis [18].

Alberti et al. also showed that the natural course of chronic HP is comparable to that of IPF. Thus, over 2 years of follow-up, mortality in the patients with IPF was higher compared with the patients with chronic HP (*p* = 0.027), although the analysis of Cox’s proportional risks did not reveal significant differences in mortality. At the same time, the groups of patients with chronic HP and IPF did not differ significantly in the rate of FVC decline [19].

The predominance of the fibrotic phenotype (91.8%) of chronic HP in our study explains the high prevalence of the progressive course of HP (30.3%) and the marked mortality (14.5%).

A limited number of studies have been devoted to predictors of a response to therapy, progression, and mortality in chronic HP. Their results are quite heterogeneous and cover a fairly wide range of parameters that should be considered in the management of patients with chronic HP [4,14,20,21].

According to the results of our study, some characteristics of patients (male sex, Charlson index, presence of diabetes mellitus, history of smoking, severity of dyspnea, presence of finger clubbing, and GAP scale) had a significant impact on the risk of progression and death of patients with chronic HP.

The effect of age and male sex on mortality from chronic HP is expected, because they are typical risk factors for many ILDs [14,22]. At the same time, it should be taken into account that the influence of these factors may also be due to the contribution of comorbid diseases. For example, Clarson et al. have shown that men with ILDs are at risk of developing coronary heart disease and myocardial infarction, which can significantly affect their prognosis [23]. The impact of comorbidity on the prognosis of patients with chronic HP has been demonstrated in several studies, which showed that patients with HP had a significantly higher Charlson comorbidity index compared with healthy controls, and a higher risk of death (HR 1.98, CI 1.61–2, 58) [2,14].

In our study, diabetes mellitus was one of the prognostic indicators for patients with chronic HP. Hyldgaard et al. also demonstrated that diabetes was a factor associated with a significantly higher mortality in IPF patients (HR 2.5, 95% CI 1.04–5.9) [24]. On the other hand, Kim et al. found no difference in survival between IPF patients with and without diabetes, although patients with IPF and diabetes had a significantly higher incidence of hypertension, cardiovascular disease, and malignancies than patients without diabetes [25]. Further studies are needed to fully clarify the impact of diabetes on prognosis in chronic HP patients.

The effect of smoking on the course of HP is quite controversial, because, on the one hand, smoking reduces the risk of developing HP, but on the other hand, smoking in patients with HP increases the risk of fibrosis and, therefore, the disease progression, due to switching the immune response to the Th2 pathway instead of Th1 and Th17 [26,27].

The GAP scale has proven to be a convenient tool for assessing the prognosis in patients with ILD, primarily with IPF. Ryerson et al. also demonstrated good reproducibility and predictive value of this scale in patients with chronic HP [28]. In the present study, we also demonstrated that a GAP score greater than 4 points can serve as one of the predictors of disease progression in chronic HP.

We found that the most important prognostic factors for chronic HP are a DLco less than 50% predicted, an SpO2 at the end of 6-MWT less than 85%, and an RA ≤ 18 mm^2^. 

Our findings are consistent with the data of the study by Lama et al., who showed that in patients with idiopathic interstitial pneumonias (IIPs), desaturation in 6-MWT was associated with an increased risk of death (hazard ratio (HR) 4.2, 95% CI 1.40–12.56) after adjusting for age, sex, smoking, baseline DLco, FVC, and resting saturation [29].

Only limited data are available on right ventricular (RV) function, RA area, and prognosis of patients with ILDs. Amano et al. demonstrated that systolic excursion in the plane of the tricuspid annulus (HR 0.85, 95% CI 0.74–0.97) and mean pulmonary artery pressure/cardiac output (HR 1.50, 95% CI 1.08–2.09) were independent predictors of all-cause mortality in patients with IIPs [30]. Rivera-Lebron et al. also found that the ratio of RV to left-ventricular diameter (HR 4.5, 95% CI 1.7–11.9), moderate to severe RA dilatation (HR 2.9, 95% CI 1.4–5.9), and RV dilatation (HR 2.7, 95% CI 1.4–11.9), respectively, were associated with an increased risk of death in patients with IPF [31]. Zhu et al. proposed to include the parameters of RV dysfunction in the GAP score to further improve its predictive value [32]. Our results also showed that the RA area had a significant prognostic value for patients with chronic HP. 

Finger clubbing was an independent predictor of survival for IPF patients in a study by Cai et al. [33]. In this study, IPF patients with finger clubbing had a longer disease duration, more frequent reticular opacities and honeycombing, and worse DLco than those without clubbing. Similarly, we have identified the prognostic role of finger clubbing for patients with chronic HP.

Other studies have paid much more attention to the impact of HRCT patterns and histological signs of fibrosis on the course and prognosis of chronic HP. According to Wang et al., independent predictors of mortality or referral for lung transplantation in patients with chronic HP were the presence of fibroblastic foci and evidence of fibrosis on histological examination [34]. Mooney et al. demonstrated that the presence of reticular changes in HRCT and auscultatory crackles is a predictor of worse prognoses in HP [21]. 

Ojanguren et al. showed that the most significant predictors of mortality or referral for lung transplantation of patients with chronic HP were age (OR 1.04, 95% CI 1.01–1.06), BAL lymphocytosis (OR 0.97, 95% CI 0, 96–0.99), DLco % predicted (OR 0.96, 95% CI 0.94–0.97), the presence of honeycombing (OR 1.75, 95% CI 1.06–2.87), and histological UIP pattern (OR 2.26, 95% CI 1.19–4.27) [4]. According to Lewandowska et al., predictors of a good prognosis were a VCmax > 65% predicted and a TLC > 72.5% predicted, and predictors of a poor prognosis were signs of fibrosis at HRCT [20].

The results of the first meta-analysis of the risk factors for death in patients with chronic HP were recently published, covering 3077 patients from 21 studies [14]. According to the data obtained with multivariate analysis, the following predictors of poor prognosis in chronic HP were identified: age (overall OR 1.036, 95% CI 1.025–1.046), male gender (overall OR 1.396, 95% CI 1.004–1.943), honeycombing on HRCT (overall OR 1.121, 95% CI 1.070–1.175), and traction bronchiectasis on HRCT (overall OR 1.107, 95% CI 1.048–1.169) [14].

Adegunsoye et al. divided various ILDs into phenotypes according to the results of cluster analysis, and showed that a DLco less than 50% predicted was a factor of an unfavorable prognosis. The worst prognosis was in patients with a relatively preserved FVC (more than 60% predicted) but a significant decrease in DLco (less than 50% predicted), predominantly male, smokers, with a greater prevalence of honeycombing [35]. The clinical significance of DLco is also emphasized by the fact that a number of studies have shown that immunosuppressive therapy in chronic HP has a significant effect on the dynamics of DLco but not on FVC [36,37].

Our study has limitations that are important to be emphasized. First, our study was retrospective, with data entered by physicians from various clinical centers, which could lead to technical errors and missing data, especially for lung function tests and echocardiography. Second, another limitation of our study is the lack of BAL cytological data, because a number of studies have indicated the possibility of using lymphocytosis in BAL as a prognostic factor [20,38,39]. The third limitation is the lack of clear information about the timing and regimens of therapy and the antigen avoidance, which made it impossible to assess the effect of therapy on the course and prognosis in patients with chronic HP. In addition, a limitation of this study is a relatively short follow-up period for patients, which limited the assessment of mortality.

## 5. Conclusions

The present study included patients with chronic HP, predominantly with a fibrotic phenotype (91.8%), which led to a high prevalence of disease progression (30.3%), low efficacy of therapy, and high mortality rates. The most significant predictors of progression and mortality were a baseline DLco < 50% predicted, an SpO2 at the end of 6-MWT < 85%, and a GAP score ≥ 4 points. Early detection of the predictors of an adverse prognosis of chronic HP is necessary for the timely initiation of antifibrotic therapy.

## Figures and Tables

**Table 1:** Baseline characteristics of patients included in the study (*n* = 292).

Characteristic	Value
Age, years	65 (58–71)
Gender (M/F), n (%)	133 (43.8%)/159 (52.3%)
BMI, kg/m^2^	28.0 (24.9–31.6)
Smoking history, pack/years	20 (13–32)
Time from symptom onset to diagnosis, months	11 (3–33)
Fibrotic phenotype, n (%) Nonfibrotic phenotype, n (%)	279 (91.8%) 13 (4.3%)
Known history of exposure, n (%)	46 (15.1%)
GAP score, points	3 (2–4)
GAP, stages (%) I II III	52.6% 42.1% 5.3%
Cough, n (%)	223 (73.4%)
Dyspnea, n (%)	263 (86.5%)
Dyspnea mMRC score, points	2 (2–3)
General weakness, n (%)	190 (62.5%)
Chest pain, n (%)	33 (10.9%)
Respiratory rate, min^−1^	20 (18–22)
Heart rate, min^−1^	85 (77–94)
SpO_2_, %	94 (90–96)
Cyanosis, n (%)	93 (17.2%)
Peripheral edema, n (%)	32 (5.9%)
Finger clubbing, n (%)	117 (21.6%)
Comorbidities: Arterial hypertension, n (%) Cardiovascular diseases, n (%) Pulmonary hypertension, n (%) GERD, n (%) Diabetes mellitus, n (%) COPD, n (%) Chronic kidney diseases, n (%) Liver diseases, n (%) Thrombosis, n (%) Oncological diseases, n (%)	166 (54.6%) 108 (35.5%) 55 (18.1%) 54 (17.8%) 44 (14.5%) 8 (2.6%) 14 (4.6%) 10 (3.3%) 8 (2.6%) 8 (2.6%)
Charlson Comorbidity Index, points	3 (2–4)
Hemoglobin, g/L	142 (130–156)
Erythrocytes, 10^12^/L	4.74 (4.33–5.2)
Platelets, 10^9^/L	233 (193–286)
Leukocytes, 10^9^/L	9.4 (7.4–12.1)
Lymphocytes, 10^9^/L	2.3 (1.7–3.1)
Monocytes, 10^9^/L	1.68 (1.13–2.4)
Neutrophils, 10^9^/L	6.0 (4.5–8.3)
Neutrophils to Lymphocytes ratio	2.7 (1.8–3.8)
ESR, mm/h	16 (8–31)
CRP, mg/L	5.0 (2.8–12.9)
FVC, L	2.2 (1.7–2.7)
FVC, % pred	67.0 (57.0–78.0)
FEV_1_, L	1.8 (1.3–2.3)
FEV_1_, % pred	67.0 (57.0–78.0)
FEV_1_/FVC, %	87.3 (81.3–92.4)
TLC, L	4.0 (3.2–4.8)
TLC, % pred	64.3 (54.5–74.5)
DLco, % pred	51.2 (41.6–59.8)
RA area, cm^2^	17.1 (13.3–19.8)
SPAP, mm Hg	36 (29–43)
TAPSE, mm	23 (18–27)
Distance 6-MWT, m	450 (390–500)
Pre-SpO_2_ in 6-MWT	95 (92–96)
Post-SpO_2_ 6-MWT	86 (81–90)
PaO_2_, mmHg	59.8 (51.8–70.1)
PaCO_2_, mmHg	33.5 (29.2–39.7)
Treatment	
Long-term oxygen therapy, n (%)	61 (20.1%)
Antifibrotic therapy, n (%)	38 (11.5%)
SCS, n (%)	125 (41.1%)
Immunosuppressants, n (%)	32 (4.6%)
Follow-up	
Progression	92 (30.3%)
Mortality	44 (14.5%)

BMI, body mass index; GERD, gastroesophageal reflux disease; COPD, chronic obstructive pulmonary disease; ESR, erythrocyte sedimentation rate; CRP, C-reactive protein; FVC, forced vital capacity; FEV_1_, forced expiratory volume in 1 s; TLC, total lung capacity; RA, right atrium; SPAP, systolic pulmonary artery pressure; 6-MWT, 6 min walking test; HRCT, high-resolution computed tomography; SCS, systemic corticosteroids.

**Table 2:** Comparative characteristics of patients with progressive and nonprogressive phenotype of chronic HP.

Characteristic	Nonprogressive Phenotype (n = 200)	Progressive Phenotype (n = 92)	*p* Value
Age, years	65.0 (58.0–70.5)	64.5 (59.3–71.8)	0.32
Gender (M/F), n (%)	83 (41.5%)/117 (58.5%)	50 (54.3%)/42 (45.7%)	0.04
BMI, kg/m^2^	28.2 (24.8–31.8)	27.8 (25.3–31.1)	0.47
Smoking history, pack/years	20 (9–30)	30 (19–40)	0.003
Time from symptom onset to diagnosis, months	11 (3–32)	12 (5–35)	0.41
Fibrotic phenotype, n (%)	187 (93.5%)	92 (100%)	
Known history of exposure, n (%)	32 (16.0%)	14 (15.2%)	0.89
GAP score, points	3 (2–4)	4 (3–5)	0.02
GAP, stages (%) I II III	61.4% 36.4% 2.3%	40.6% 50.0% 9.4%	
Dyspnea mMRC, points	2 (2–3)	2 (2–3)	0.94
Cough, n (%)	149 (74.5%)	74 (80.4%)	0.27
General weakness, n (%)	135 (67.5%)	55 (59.8%)	0.19
Chest pain, n (%)	21 (10.5%)	12 (13%)	0.52
Respiratory rate, min^−1^	20 (18–22)	21 (18–22)	0.87
Heart rate, min^−1^	84 (77–92)	88 (75–100)	0.15
Cyanosis, n (%)	65 (23.5%)	29 (32.5%)	0.61
Peripheral edema, n (%)	20 (10%)	8 (8.7%)	0.06
Finger clubbing, n (%)	73 (36.5%)	44 (47.8%)	0.09
Comorbidities: Arterial hypertension, n (%) Cardiovascular diseases, n (%) Pulmonary hypertension, n (%) GERD, n (%) Diabetes mellitus, n (%) COPD, n (%) Chronic kidney diseases, n (%) Liver diseases, n (%) Thrombosis, n (%) Oncological diseases, n (%)	105 (53%) 70 (35%) 32 (16%) 38 (19%) 22 (11%) 6 (3%) 8 (4%) 7 (3.5%) 5 (2.5%) 2 (1%)	61 (66.3%) 38 (41.3%) 23 (25%) 16 (17.4%) 22 (23.9%) 2 (2.2%) 6 (6.5%) 3 (3.3%) 3 (3.3%) 6 (6.5%)	0.03 0.3 0.07 0.74 0.004 0.69 0.35 0.92 0.71 0.01
Charlson comorbidity index, points	3 (2–4)	3 (3–5)	0.04
Hemoglobin, g/L	142 (129–156)	142 (131–153)	0.74
Erythrocytes, 10^12^/L	4.7 (4.3–5.2)	4.8 (4.4–5.2)	0.49
Platelets, 10^9^/L	242 (200–285)	217 (177–288)	0.06
Leukocytes, 10^9^/L	9.6 (7.7–12.5)	9.1 (7.2–11.7)	0.25
Lymphocytes, 10^9^/L	2.3 (1.6–3.1)	2.3 (1.8–3.2)	0.21
Neutrophils to lymphocytes ratio	2.8 (1.8–4.0)	2.3 (1.7–3.4)	0.12
Monocytes, 10^9^/L	1.5 (0.9–2.4)	1.9 (1.2–2.5)	0.27
Neutrophils, 10^9^/L	6.4 (4.7–8.5)	5.9 (3.6–7.7)	0.14
ESR, mm/h	14 (8–25)	24 (9–37)	0.04
CRP, mg/L	5.3 (2.9–14.5)	5 (2.5–12.0)	0.68
FVC, L	2.2 (1.7–2.7)	2.1 (1.7–2.6)	0.57
FVC, % pred	67 (57–82)	66.0 (57.0–74.5)	0.20
TLC, L	4.0 (3.2–4.9)	3.7 (2.9–4.6)	0.43
TLC, % pred	64.7 (60.0–84.5)	62.1 (54.9–71.4)	0.96
DLco, % pred	55.0 (45.0–65.0)	44.5 (35.1–53.2)	0.0001
SPAP, mmHg	35 (29–43)	38 (29–43)	0.46
TAPSE, mm	27.5 (19.8–43.0)	21 (18–24)	0.16
RA area, cm^2^	16.8 (13.5–19.2)	19 (13.0–24.9)	0.37
Distance 6-MWT, m	460 (360–550)	450 (390–493)	0.74
Pre-SpO_2_ 6-MWT	95 (93–96)	95 (92–96)	0.48
Post-SpO_2_ 6-MWT	88 (84–90)	84 (79–88)	0.001
PaO_2_, mmHg	59.8 (52.9–72.1)	59.0 (51.1–67.2)	0.48
PaCO_2_, mmHg	34.8 (28.4–40.3)	33 (29–38)	0.63
Treatment			
Long-term oxygen therapy, n (%)	38 (19.0%)	70 (76.1%)	0.0001
Antifibrotics, n (%)	18 (17.1%)	20 (24.4%)	0.01
SCS, n (%)	81 (77.1%)	44 (60.3%)	0.24
Immunosuppressants, n (%)	14 (13.3%)	18 (24.7%)	0.03
At the end of follow-up			
Dyspnea mMRC, points	3 (2–4)	3 (3–4)	0.01
FEV_1_, L	2.1 (1.6–2.5)	1.6 (1.2–2.2)	0.005
FEV_1_, % pred	65.0 (55.0–78.5)	54.2 (45.0–63.0)	0.0001
Delta FEV_1_, %	4.0 (1.0–6.0)	11.0 (10.0–13.0)	0.0001
DLco, % pred	50.5 (45.6–57.8)	32 (22.0–40.5)	0.0001
Delta DLco,%	5.0 (1.3–10.3)	14.5 (11.0–16.4)	0.0001
Distance 6-MWT, m	430 (385–515)	315 (204–366)	0.001
Delta distance 6-MWT, m	50 (33–75)	132 (103 -178)	0.0001
Pre-SpO_2_ 6-MWT	94 (92–98)	92 (89–95)	0.009
Post-SpO_2_ 6-MWT	87 (80–90)	80 (73–87)	0.02
Mortality	0	44 (47.8%)	0.0001

BMI, body mass index; GERD, gastroesophageal reflux disease; COPD, chronic obstructive pulmonary disease; ESR, erythrocyte sedimentation rate; CRP, C-reactive protein; FVC, forced vital capacity; FEV_1_, forced expiratory volume in 1 s; TLC, total lung capacity; RA, right atrium; SPAP, systolic pulmonary artery pressure; 6-MWT, 6 min walking test; HRCT, high-resolution computed tomography; SCS, systemic corticosteroids.

## Data Availability

We encourage all authors of articles published in MDPI journals to share their research data.
